# Illustration image style transfer method design based on improved cyclic consistent adversarial network

**DOI:** 10.1371/journal.pone.0313113

**Published:** 2025-01-14

**Authors:** Xiaojun Wang, Jing Jiang

**Affiliations:** College of Arts, Anhui Xinhua University, Hefei, China; New York University Abu Dhabi, UNITED ARAB EMIRATES

## Abstract

To improve the expressiveness and realism of illustration images, the experiment innovatively combines the attention mechanism with the cycle consistency adversarial network and proposes an efficient style transfer method for illustration images. The model comprehensively utilizes the image restoration and style transfer capabilities of the attention mechanism and the cycle consistency adversarial network, and introduces an improved attention module, which can adaptively highlight the key visual elements in the illustration, thereby maintaining artistic integrity during the style transfer process. Through a series of quantitative and qualitative experiments, high-quality style transfer is achieved, especially while retaining the original features of the illustration. The results show that when running on the Monet2photo dataset, when the system iterates to 72 times, the loss function value of the research method approaches the target value of 0.00. On the Horse2zebra dataset, as the sample size increases, the research method has the smallest FID value, and the value approaches 40.00 infinitely. With the change of peak signal-to-noise ratio, the accuracy of the research algorithm has been greater than 95.00%. Practical application found that the color of the image obtained by the research method is more gorgeous and the line features are more obvious. The above results all show that the research method has achieved more satisfactory results in the task of style transfer of illustration images, especially in terms of the accuracy of style transfer and the retention of image details.

## 1. Introduction

With the booming development of digital media art, style transfer technology for illustration images has gradually become a research hotspot in the field of computer vision and graphics. This technology can apply a specific artistic style to a target image to create a completely new visual work [1, 2]. In recent years, the introduction of deep learning technology has greatly promoted the development of this field, especially the emergence of generative adversarial networks (GANs), which provide a powerful learning framework for style transfer [3]. However, existing methods still face challenges in dealing with complex styles and maintaining content consistency.

In existing research, cycle-consistent adversarial networks (CycleGANs) have attracted widespread attention due to their feature of not requiring paired training samples. CycleGANs can transfer image styles between different domains while maintaining content consistency by introducing cycle consistency loss [4]. However, this method often finds it difficult to balance the quality of style transfer and the fidelity of content when dealing with illustration images with rich details [5]. In addition, some research works have tried to improve the effect of style transfer by improving the network structure, loss function or training strategy, but these methods often require a lot of experimental adjustments to adapt to different styles and content.

In view of the limitations of existing methods, this study proposes a style transfer method for illustration images based on an improved cycle-consistent adversarial network. The residual feature aggregation module (RFA) structure is introduced in the process to enhance the stability of the network and make the illustration feature conversion more complete. At the same time, the Cycle-GAN network model is improved by combining the multi-spectral channel attention module FcaNet and MS-SSIM loss, hoping to achieve a more accurate and natural illustration image style transfer effect.

The innovations of the research can be divided into the following points: (1) An RFA module is proposed in the experiment, which not only retains the features extracted from each residual block in the residual network, but also innovatively aggregates at the final output layer. This strategy significantly enhances the model’s ability to capture and express key visual features, and provides more accurate feature support for illustration style transfer. (2) In order to solve the problem of unclear style feature transfer and model collapse caused by unstable high-level abstract features, this study innovatively combined the dense connection network and Adams fast connection method in the converter. This combination not only optimizes the flow and stability of features, but also significantly improves the adaptability and migration efficiency of the model to complex style features. (3) MRAB is used to recover image detail features. In the process of style transfer, MRAB aggregates image features on different scales to effectively improve the quality of image detail recovery, making the generated illustration image more abundant and accurate while maintaining the original artistic style.

The main contributions of the study can be divided into four points: (1) The constructed method combines the precise feature extraction capability of FcaNet to conduct in-depth analysis and optimization of the detailed features in the illustration image. This solves the problem of detail loss that is often ignored by existing methods when dealing with complex illustration style transfer. (2) The improved Cycle-GAN algorithm breaks through the technical bottleneck of traditional style transfer algorithms in maintaining style consistency and content authenticity by introducing a new loss function. The MS-SSIM loss is added to the cycle consistency loss, making the pictures obtained after the converted illustrations more realistic. (3) The combination of FcaNet and the improved Cycle-GAN method provides a new perspective for the field of image style transfer. This can not only improve technical performance, but more importantly, it provides new academic ideas for how to balance diversity and fidelity in style transfer. (4) The research provides a new technical framework for the field of illustration image style transfer, which can not only promote technical progress in this field, but also provide rich experimental data and theoretical basis for future research.

## 2. Related works

Image style transfer is a technique of applying the style of one or more images to another image. Its goal is to combine the stylistic features of one or more images with the content characteristics of the target image to produce an output image with a new style. Illustration image style transfer applies an illustration style to a target image. Compared with other style transfer methods, illustration image style transfer focuses more on imitating the characteristics of hand-drawn illustrations, such as the thickness of lines, the flatness of colors, and the simplification of textures. By applying illustration image style transfer, one can transform the target image into a work of art that looks like a hand-drawn illustration, adding a unique artistic style and charm. As computer vision and machine learning technologies develop, image style transfer has become an active research field. L Wang et al. proposed a new ESA-CycleGAN model to improve loss of detail and poor visual effect in the existing style transfer methods. The model effectively captured the global and detailed features of the image through a generator, a discriminator, and an edge feature extraction network. Tests on four datasets showed that the model preserved details better in style delivery [6]. To avoid errors during image reconstruction, G Wang et al. proposed a double-loop constrained cross-domain Generative Adversarial Network (GAN) method for image-to-image translation. An innovative long-period and short-period consistent loss mechanism was introduced in the process, which reduced the accumulation of errors by applying double-cycle constraints in both short and long periods. Results showed that it was significantly better than other methods in most image transformation scenarios and was suitable for multiple datasets [7]. To extend the ability of the recurrent consensus generative adversarial network to transmit images between different image domains, researchers such as CTU et al. introduced a conditional constraint that was presented as a feature map of the target style. Experiments showed that the framework was not only feasible, but also was applied to a variety of transformation tasks, such as facial expression, facial aging, and different feature synthesis [8]. To achieve the purpose of image-to-image translation of paired images, V Bharti et al. proposed an image translation method based on Evolutionary Multi-objective CycleGAN (EMOCGAN). The model combined evolutionary computation and multi-objective optimization to avoid local optimality through metropolitan acceptance criteria and a mechanism based on Pareto selection. The results showed that the model surpassed the current leading technologies in terms of visual authenticity, background information preservation, and prominent object representation, and had a higher UQI value [9]. To make the obtained SAR image a grayscale image rich in color information, G Ji et al. proposed an image colorization method using multi-domain cyclic consistency generative adversarial network. The method enhanced the colorization effect through two strategies: one was to propose a mask vector that uses cyclic consistency loss binding without pairwise data training, and the other was to apply multi-domain classification loss to ensure the desired color of generated image. On SEN1-2 dataset, this method had significant effectiveness [10].

At the same time, many scholars have also discussed the application of the CycleGAN model in other fields. To show the texture characteristics of traditional Chinese painting, X Peng et al. proposed a landscape image style conversion method based on the contour enhancement CycleGAN framework. In the process, a contour enhancement translation branch was designed, and an edge detection algorithm was added to calculate the gradient to correctly guide the photo-to-image conversion. The results showed that the algorithm converted real-life landscape photos into target Chinese landscape paintings, and the composite similarity measure of the images was as high as 0.92 [11]. To evaluate the driving behavior of unmanned vehicles, Y Li et al. proposed an image style transfer method using structural information and generative adversarial networks. The framework was used to analyze road scenarios throughout the day to maintain the integrity of the information structure. The results showed that this method had significant effectiveness and performed style transfer on high-quality images [12]. To avoid the recurrence model from falling into different mode collapse states, H You et al. proposed a generative adversarial network using cyclic consistency, and tested the latent variables to maximize the performance of the optimization model. Through verification on multiple datasets, it was found that the algorithm positively improved the pixel accuracy of the image of the cityscape semantic segmentation task by 15%, and the final image definition was higher [13]. To avoid the inability to transfer image style by combining images from small datasets, Chen et al. proposed an intelligent generation method using generative adversarial networks. This method effectively enhanced user intent with prior knowledge, and at the same time generated specific natural styles. Results showed that the images local details obtained by this method were very clear, and the contrast was significantly enhanced [14]. To automate the diagnosis and identification of diabetic retinopathy, J D Bodapati et al. developed a deep learning network structure that incorporates a gated attention mechanism. By simplifying the data by using spatial pooling technology, the study effectively retained key information and avoided the loss of a large amount of information. In addition, the gated attention unit integrated in the network focused on identifying the lesion area, reducing the focus on the normal area. The hybrid model achieved a prediction accuracy of 82.54% and a Kappa score of 79, showing better performance than the single model [15].

In real world situations, accurate target dynamic models and noise statistics are often difficult to obtain, which limits the performance of traditional Kalman filters. To overcome this challenge, Jin et al. developed an adaptive state estimation technique that utilizes an attentional parameter learning module that is capable of eliminating the need to rely on precise model parameters. By using the expectation maximization algorithm to update system parameters online and combining with Kalman filter for state prediction, this method has shown higher estimation accuracy than traditional methods in experiments [16]. With the booming development of social media and e-commerce, advances in Internet technology have promoted the rapid growth of multimodal data, which has stimulated the demand for efficient cross-modal retrieval systems. Li et al. provide a comprehensive overview of cross-modal image-text retrieval that goes beyond the limitations of previous studies, which tend to focus on a single technique such as subspace learning or deep learning [17]. Chu et al. also propose an innovative two-stage robotic grab detection strategy to address the limitations of end-to-end deep learning models. The strategy first uses particle swarm optimization algorithm to generate candidate grasping points, and then filters them through CNN model to determine the best grasping location. Experimental results on the Cornell Grasp dataset show that the proposed method achieves a high accuracy of 92.8%, which proves its effectiveness and leadership in real-time operation [18]. In the field of target detection, traditional models are difficult to deploy on resource-constrained edge devices due to the large number of parameters and large resource consumption. To overcome these limitations, Yang et al. designed YOLOV8-Lite, a lightweight object detection model based on YOLOv8 architecture. Tests on NEXET and KITTI datasets show that the detection performance of YOLOv8-lite has been effectively enhanced while maintaining lightweight [19].

Through in-depth analysis of existing literature, it can be found that CNN-based style transfer methods and CycleGAN models have made significant progress in the field of image style transfer. From the original CycleGAN model to improved variants for specific problems, such as improvements for low-resolution inputs, algorithms for multi-style transfer, and optimization of the discriminator and generator in generative adversarial networks (GAN), researchers have Various methods have been explored to improve the quality and efficiency of style transfer. However, most methods still have certain challenges when processing illustration images with high details and complex textures, and it is difficult to achieve satisfactory results. In order to better solve the above problems, the experiment proposes an illustration image style transfer method based on an improved cycle consistency adversarial network. Study how to improve the quality and efficiency of style transfer by further optimizing the model and loss function; and combine deep learning with other image processing technologies to expand the scope of application of the algorithm. It is expected to reveal the characteristics of illustration image style transfer and promote illustration image style. Migration technology moves forward.

## 3. Illustration image style transfer method by integrating FcaNet and improving CycleGAN network

In the field of image style transfer, CycleGAN has been proven to be an effective unsupervised learning method, especially when pairs of samples are not required. However, when applied to the style transfer of illustrated images, CycleGAN faced the challenge of capturing and preserving the details of a complex art style. In order to improve style transfer quality, especially to achieve a high degree of style consistency while maintaining the details and texture of illustration images, this paper proposes an illustration image style transfer method that integrates FcaNet and improved CycleGAN network.

### 3.1. Illustration image style transfer model based on CycleGAN

**Fig 1:**
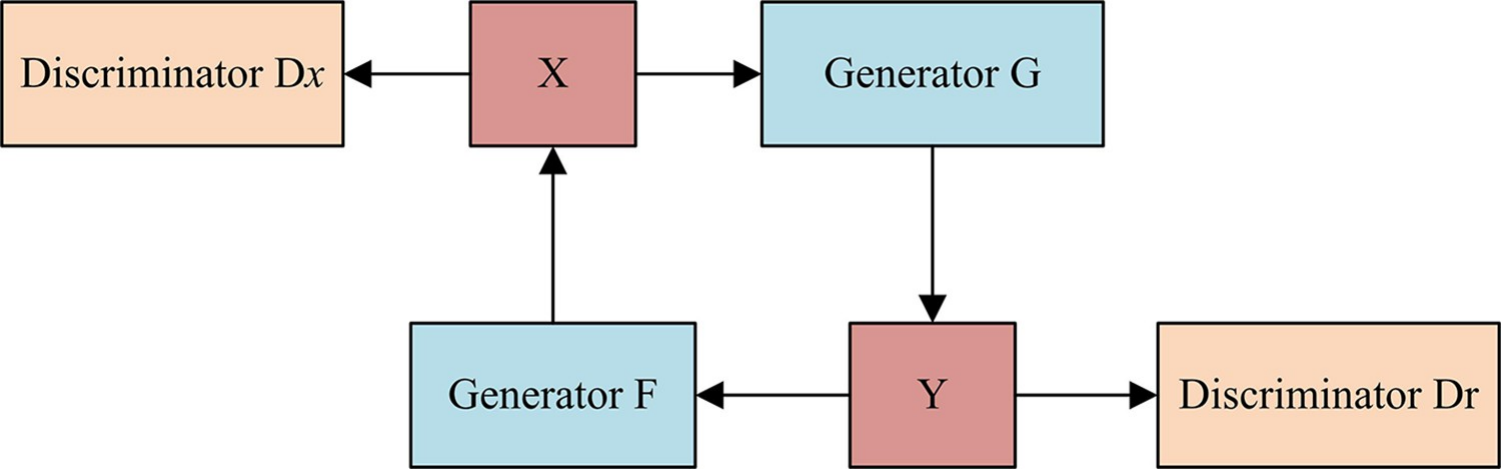
Network structure of CycleGAN model.

The goal of image style transfer is to combine the stylistic features of one or more images with the content features of the target image to generate an output image with a new style. Illustration image style transfer pays more attention to imitate the characteristics of hand-drawn illustration, such as the thickness of lines, the flatness of colors and the simplification of textures. By applying illustration image Style transfer, you can transform the target image into a work of art that looks like a hand-drawn illustration, adding a unique artistic style and charm. Illustration image style transfer can be achieved by using a pre-trained neural network model that learns the illustration style features and is able to apply these features to the target image, resulting in an output image with an illustration style [20]. This technique has been widely used in the fields of artistic creation, design, and entertainment [21, 22]. Generally speaking, Pix2Pix and CycleGAN models are commonly used in image style transfer models. Among them, CycleGAN is a cross-domain image style transfer model, the biggest feature of which is unsupervised learning, and the model solves the problem of training images in pairs when training the network by introducing the cyclic consistency loss function. The CycleGAN network is shown in Fig 1.

**Fig 2:**
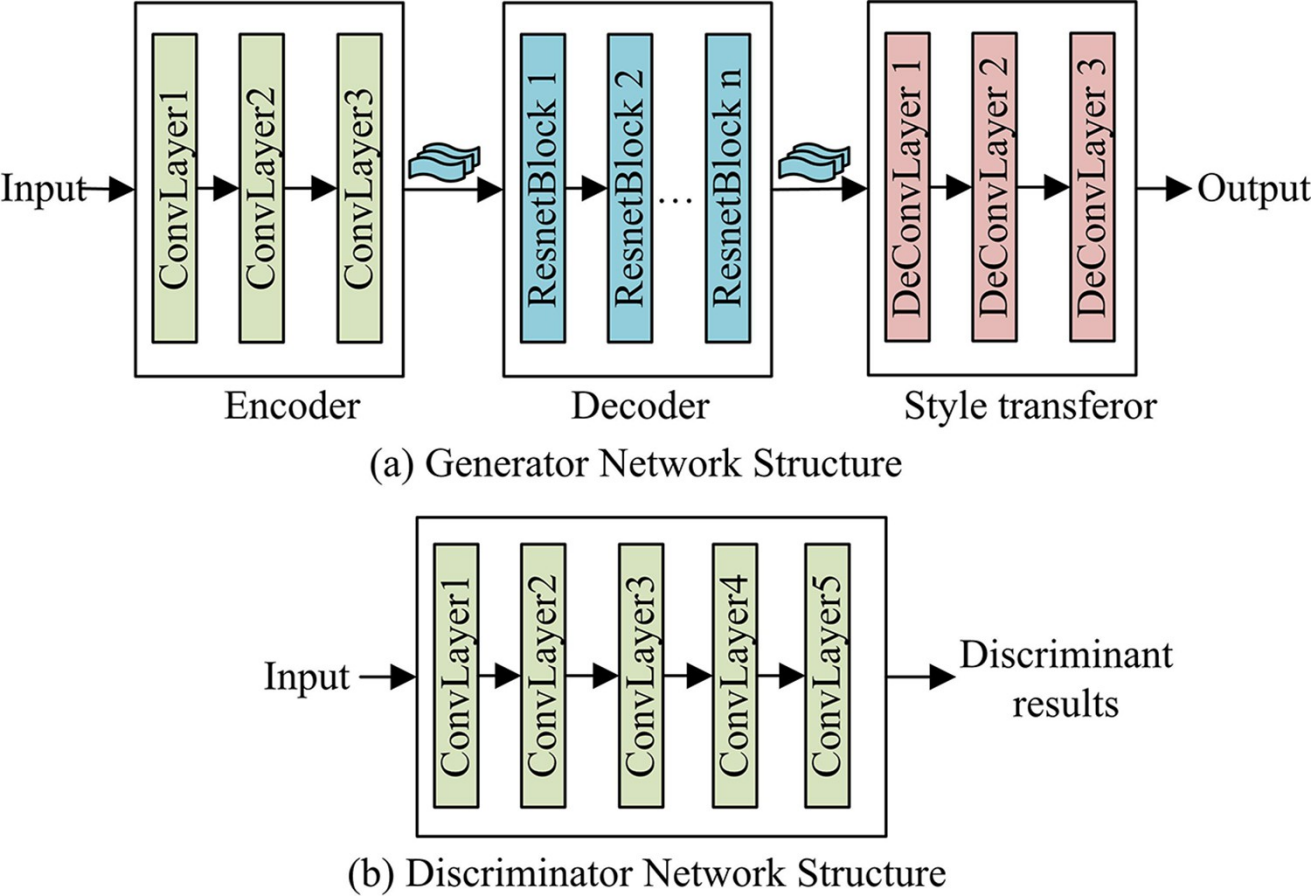
Generator network and discriminator network.

CycleGAN’s generator network consists of three parts: encoder, style converter and decoder. The encoder extracts image features by a three-layer convolutional network, while the style conversion migrator contains a three-layer residual network, which can effectively improve gradient vanishing in the generative network and improve the actual generative network performance. The decoder contains three deconvolution layers and is responsible for recovering low-level features from feature vectors and generating images. These network structures are important in image style transfer, transforming and migrating images of different styles [23]. Their design and training methods make image style transfer more flexible and efficient. The generator network and discriminator network are shown in Fig 2.

When the generator *G* is trained, the domain *X* is the source domain and the domain *Y* is the target domain. At this time, the discriminator *D*_*Y*_ should judge the true or false situation of *y* and *G*(*x*), and *G* and *D*_*Y*_ are used to deduce the mapping loss function between the two in Eq (1).


$$L_{GAN}(G_{X\to Y},D_{Y})=E_{y∼P_{data}(y)}\log D_{Y}(y)+E_{x∼P_{data}(y)}\log(1-D_{Y}(G_{X\to Y}(x)))$$ (1)

In addition, when the generator *F* starts training, the resulting source domain is *Y* and the target domain is *X*. The corresponding loss function between the *F* and the *D*(*x*) is calculated in Eq (2).


$$L_{GAN}(F_{Y\to X},D_{X})=E_{x∼P_{data}(x)}\log D_{X}(x)+E_{y∼P_{data}(y)}\log(1-D_{X}(F_{Y\to X}(y)))$$ (2)

When the images in *X* are converted to *Y*, the matching distribution images can be found in the source domain, but there is no guarantee that *Y* most of the images in the domain *X* will not appear corresponding to a small part of the images in the domain *Y*. This situation can lead to the model not being able to learn sufficiently, resulting in meaningless mappings [24, 25]. In order to avoid this kind of mapping, the experiment intends to add constraints when training the generators *G* and *F* to meet the cyclic consistency. After converting an image *x* in *X* to *Y*, it is then transferred back to an image *x*, and for each image in the domain *Y*, the generators *G* and *F* establish a good correspondence under certain conditions between the source domain and the target domain. The relationship between the two is defined in Eq (3).


$$\left\{ \begin{array}{l} x\to G(x)\to F(G(x))=x\\ y\to G(y)\to F(G(y))=y \end{array}\right.$$ (3)

The purpose of introducing circular consistency is to avoid all images in the source domain *X* being mapped to the same image in *Y*, which reduces the built model crashing risk. The loss function of cyclic consistency can then be defined by the relationship between the two, as shown in Eq (4).


$$L_{CCL}(G_{X\to Y},F_{Y\to X})=E_{y∼P_{data}(y)}({\left\|G_{X\to Y}(F_{Y\to X}(y)-y)\right\|}_{1})+E_{x∼P_{data}(y)}({\left\|F_{Y\to X}(G_{X\to Y}(x)-x)\right\|}_{1})$$ (4)

In Eq (4), *L*_*CCL*_ represents the loss function. Summarizing the above equation, we can obtain the objective function of the cyclic consensus adversarial network, which is calculated in Eq (5).


$$L^{\prime }(G_{X\to Y},F_{Y\to X})=\varepsilon_{CCL}L_{CCL}(G_{X\to Y},F_{Y\to X})+L_{GAN}(F_{Y\to X},D_{X})+L_{GAN}(G_{X\to Y},D_{Y})$$ (5)

**Fig 3:**
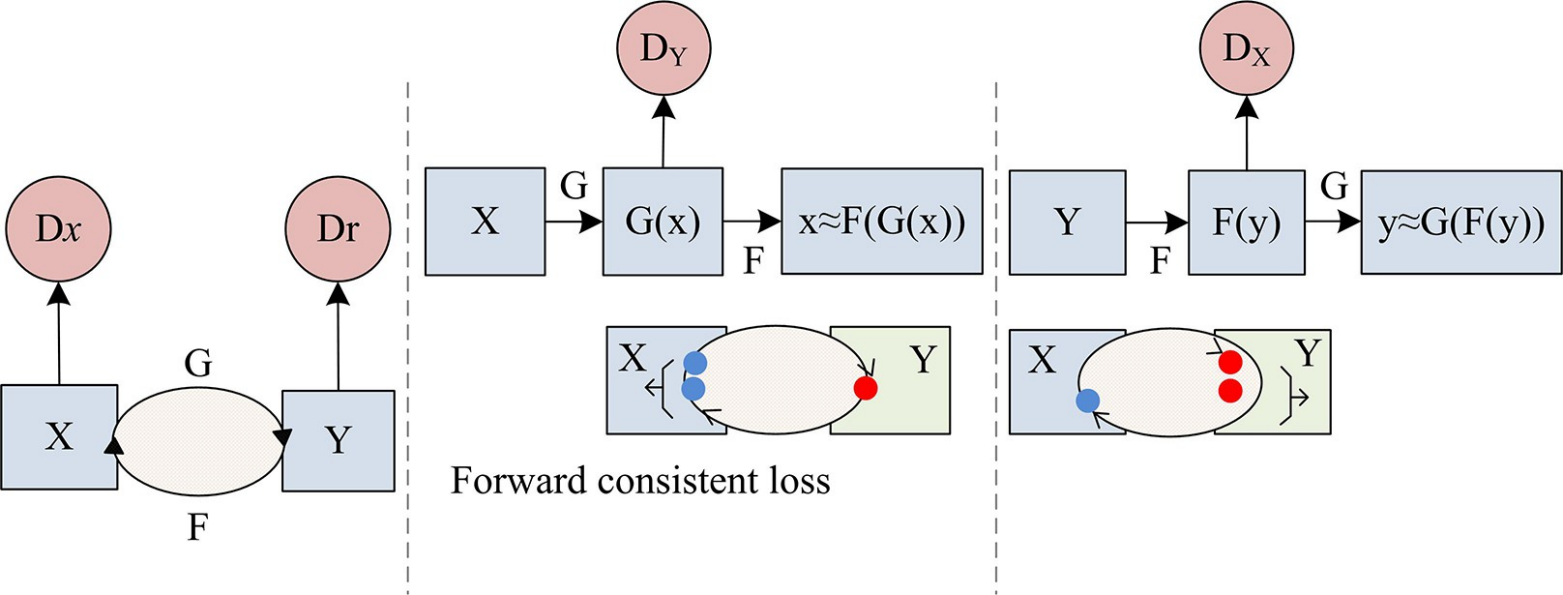
CycleGAN cycle consistency.

In Eq (5), *L*′ represents the objective function and the *ε*_*CCL*_ represents the parameter that adjusts the weight of the loss function in the objective function. CycleGAN cyclic consistency is shown in Fig 3.

### 3.2. Illustration image style transfer method using RFA-CycleGAN

In the task of style transfer, especially when dealing with highly stylized illustrated images, CycleGAN is often difficult to capture and retain details, resulting in a lack of realism and artistry in the transfer results. In addition, these models are prone to pattern crashes when dealing with unpaired data, i.e. the resulting images are not visually consistent with the target style. The attention mechanism is introduced to solve these problems. By focusing on the key visual areas and style features in the image, the model can improve its ability to capture details, so as to better retain the artistic characteristics of the original illustration in the process of style transfer. The cyclic consistency adversarial network, combined with the attention mechanism, can learn the complex mapping relationship between the source domain and the target domain more effectively, reduce the risk of pattern collapse, and improve the accuracy of style transfer [26, 27]. In general, the introduction of the RFA structure has brought substantial improvements to the CycleGAN model, especially in terms of feature transfer, network depth management, detail feature retention, image quality improvement, and enhanced generalization capabilities. These improvements enable the constructed model to provide better performance and results when dealing with complex illustration image style transfer tasks. The main purpose is to not only complete the image style transfer of illustrations, but also enhance the stability of the network, and make the feature transformation more sufficient, so as to meet the needs of the style migration of illustration images. Firstly, the class residual model is used to learn and pass the input information. When constructing a residual-like CNN model, the model can be regarded as a multi-level complex mapping. The layer iterative calculation of the conventional residual network is obtained in Eq (6).


$$h_{t+1}=h_{t}+f(h_{t},\theta_{t})$$ (6)

In Eq (6), *h*_*t*+1_ represents a layer iteration, *t* represents a certain layer of the residual network, and *t* represents an iterative solution of a differential equation, with values range of (0,*T*), assuming more network layers and smaller step sizes, which can be optimized to obtain a new solution equation as shown in Eq (7).


$$\frac{dh(t)}{dt}=f(h(t),t,\theta )$$ (7)

Then, the experiments combine with the solution of the initial value problem of the residual-like network, and the obtained differential equation is discretized to establish the recursive equation for the numerical solution, and the differential equation is used to find the initial value. The Adams method is used as a method to solve the initial value, and the calculation of the initial value is shown in Eq (8).


$$y_{n+k}=y_{n+k-1}+h\sum_{i=0}^{k}\beta_{i}f_{n+i}$$ (8)

At the same time, combined with the Adams method, a residual-like network with the properties of the Adams method can be constructed, and finally the later oscillation phenomenon of the model can be effectively suppressed. However, an important feature of the current deep neural network is that it has added shortcut connections, which makes the shortcuts in the network more in-depth, and can learn more abstract features from them, so as to learn the connection between different features more efficiently. In Dense Convolutional Networks (DenseNet), the association between different convolutional layers is usually achieved through shortcut connections within the same module, and each layer receives the output of all the layers before it in the module, thereby enhancing the connection between different features [28, 29]. See Eq (9) for specific calculations.


$$x_{1}=H_{l}(\left[x_{0},x_{1},x_{2},\cdots ,x_{l-1}\right])$$ (9)

**Fig 4:**
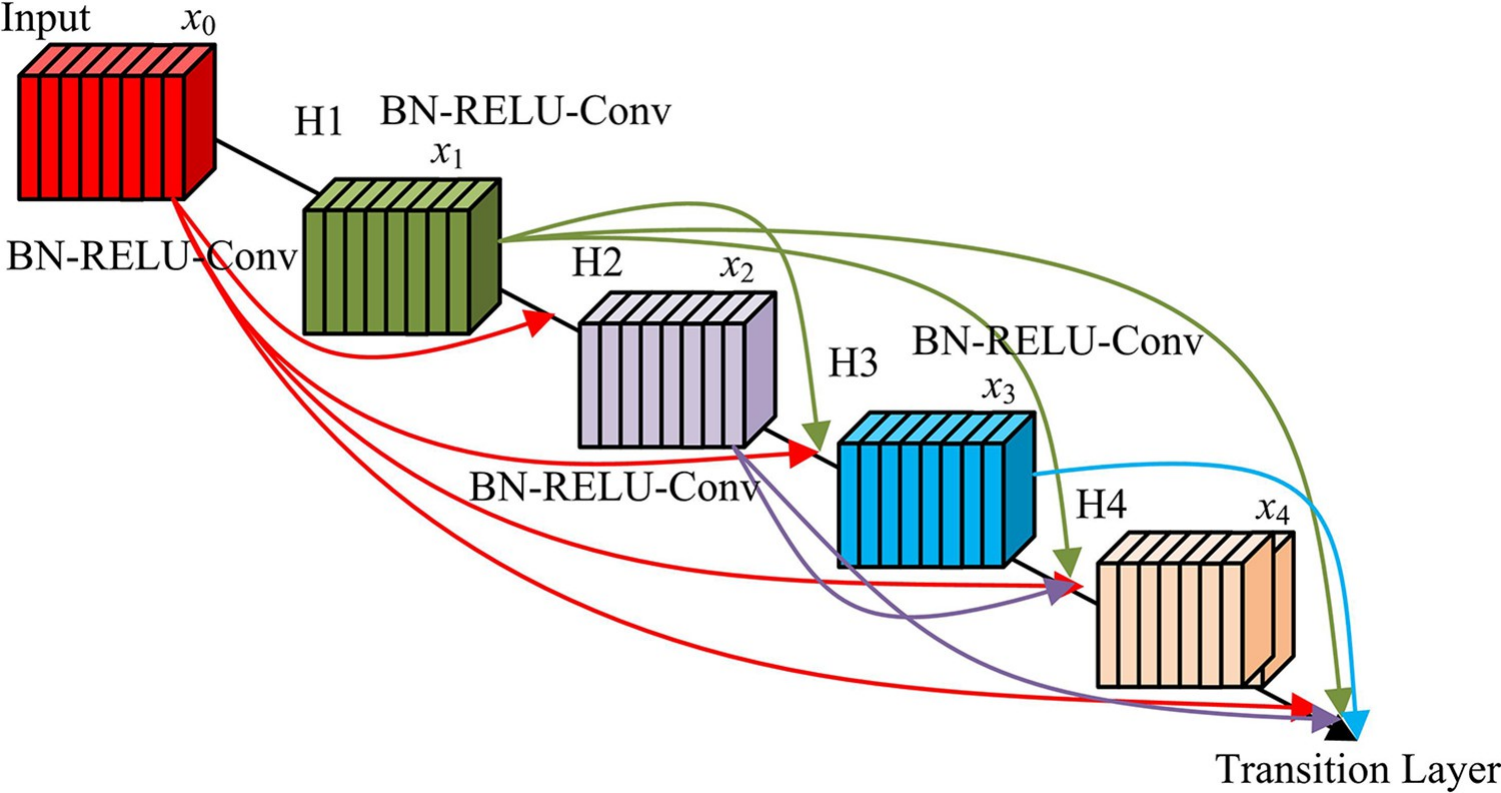
Densely linked network.

In Eq (9), $\left[x_{0},x_{1},x_{2},\cdots ,x_{l-1}\right]$ represents the method in which the output feature maps are stitched together. The obtained DenseNet structure is slenderer, which reduces the network parameters. The specific structure is shown in Fig 4.

The stability of the image reconstruction results obtained by the above structure is largely affected by the high-level abstract features. Therefore, in the style converter, the paper designs Adams dense link blocks to connect feature transformations [30, 31]. Considering the limitations of the computer system, the Adams method of experimental design is constructed and set using the second-order explicit equation, *k* = 2, *β*_*i*_ =2, and the initial value is calculated as shown in Eq (10).


$$h_{n+2}=h_{n+1}+k_{n}f_{n}+(1-k_{n})f_{n+1}$$ (10)

**Fig 5:**
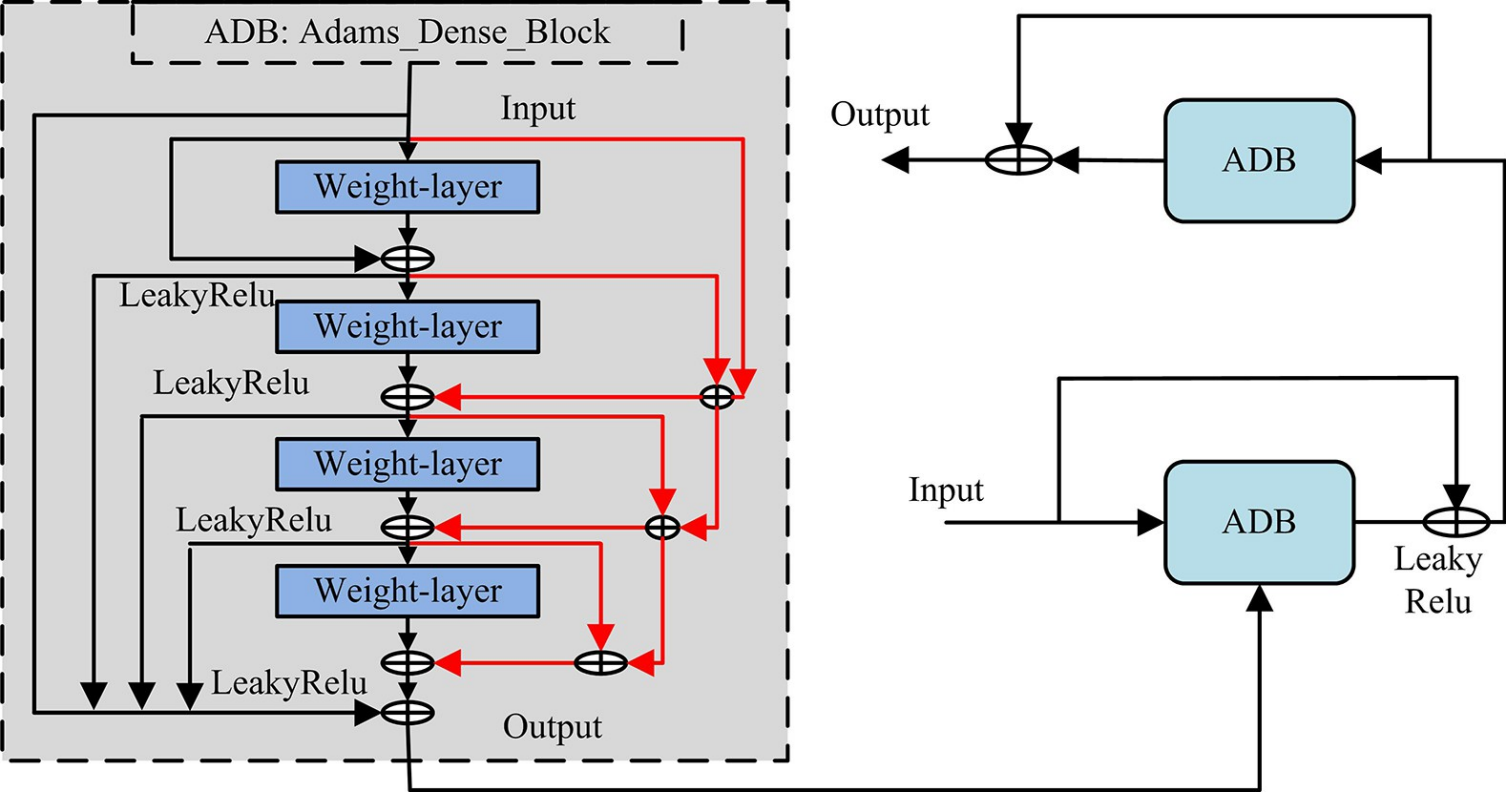
Adams dense link module.

Finally, the operation of the Adams dense connection block combined with the Adams method and the dense connection network design is shown in Fig 5.

### 3.3. Illustration image style transfer model based on improved CycleGAN

The improved Cycle-GAN network model can effectively improve the efficiency of image migration model, but the actual generated images still have problems such as target detection failure and poor detail processing. In order to solve the above problems, this paper proposes an improved Cycle-GAN network model based on the loss of multi-spectral channel attention modules FcaNet and MS-SSIM. The introduction of attention mechanism into the information processing of computer vision can effectively allocate limited computing resources to important objects and produce results consistent with human visual cognition [32, 33]. The U-Net network adopts a quadruple attention mechanism, and captures the cross-dimensional information interaction between space and channel position through four branches. However, due to the limitations of computer system overhead, the most critical step in the channel attention method is to perform scalar calculations for each channel. For this purpose, the Global Average Pooling (GAP) operation is often used. GAP has become a standard choice in the field of deep learning due to its simplicity and high efficiency [34]. FcaNet treats scalars in attention channels as a compression problem, that is, scalar compression encoding is used to encode the information in the channel, and the overall representation of the channel is maintained as much as possible. Among them, the Discrete Cosine Transform (DCT) has strong energy compression characteristics, which can obtain high-quality data compression ratio during operation. The basis function expression of a classical 2D DCT is shown in Eq (11).


$$\left\{ \begin{array}{l} f_{h,w}^{2d}=\sum_{i=0}^{H-1}\sum_{j=0}^{W-1}x_{i,j}^{2d}B_{h,w}^{i,j}\\ s.t.i\in \{0,1,\cdots ,H-1\},j\in \{0,1,\cdots ,W-1\} \end{array}\right.$$ (11)

In Eq (11), *f*^2*d*^ represents the 2d DCT spectrum, *f*^2*d*^∈*R*_*H*×*W*_. *x*^2*d*^ represents the input, *x*^2*d*^∈*R*_*H*×*W*_. *H* represents *x*^2*d*^ ‘s height. *W* represents *x*^2*d*^ ‘s width. In the multi-spectral channel attention network, the input features are first divided into many parts along the channel dimension, and the corresponding DCT frequency components can be assigned to each part, and the *Freq* vectors of different parts are obtained according to the assigned DCT frequency components. The specific calculation is shown in Eq (12).


$$\left\{ \begin{array}{l} Freq^{i}=2DDCT^{u_{i},v_{i}}(X^{i})=\sum_{h=0}^{H-1}\sum_{w=0}^{W-1}X_{h,w}^{i}B_{h,w}^{u_{i},v_{j}}\\ s.t.i\in \{0,1,\cdots ,n-1\} \end{array}\right.$$ (12)

In Eq (12), a [*u*_*i*_,*v*_*i*_] represents two-dimensional exponent of frequency component corresponding to *X*_*i*_. After exponential compression, *Freq*_*i*_ can be converted into a *C*′ dimension vector, and the computational equation is obtained as shown in Eq (13).


$$Freq=cpmpress(X)=cat(\left[Freq^{0},Freq^{1},\cdots ,Freq^{n-1}\right])$$ (13)

In Eq (13), *Freq* represents the resulting polyspectral vector, *Freq*∈*RC*. Based on the above, the complete multispectral channel attention network structure is calculated in Eq (14).


ms.att=sigmoid(fc(Freq)) (14)

**Fig 6:**
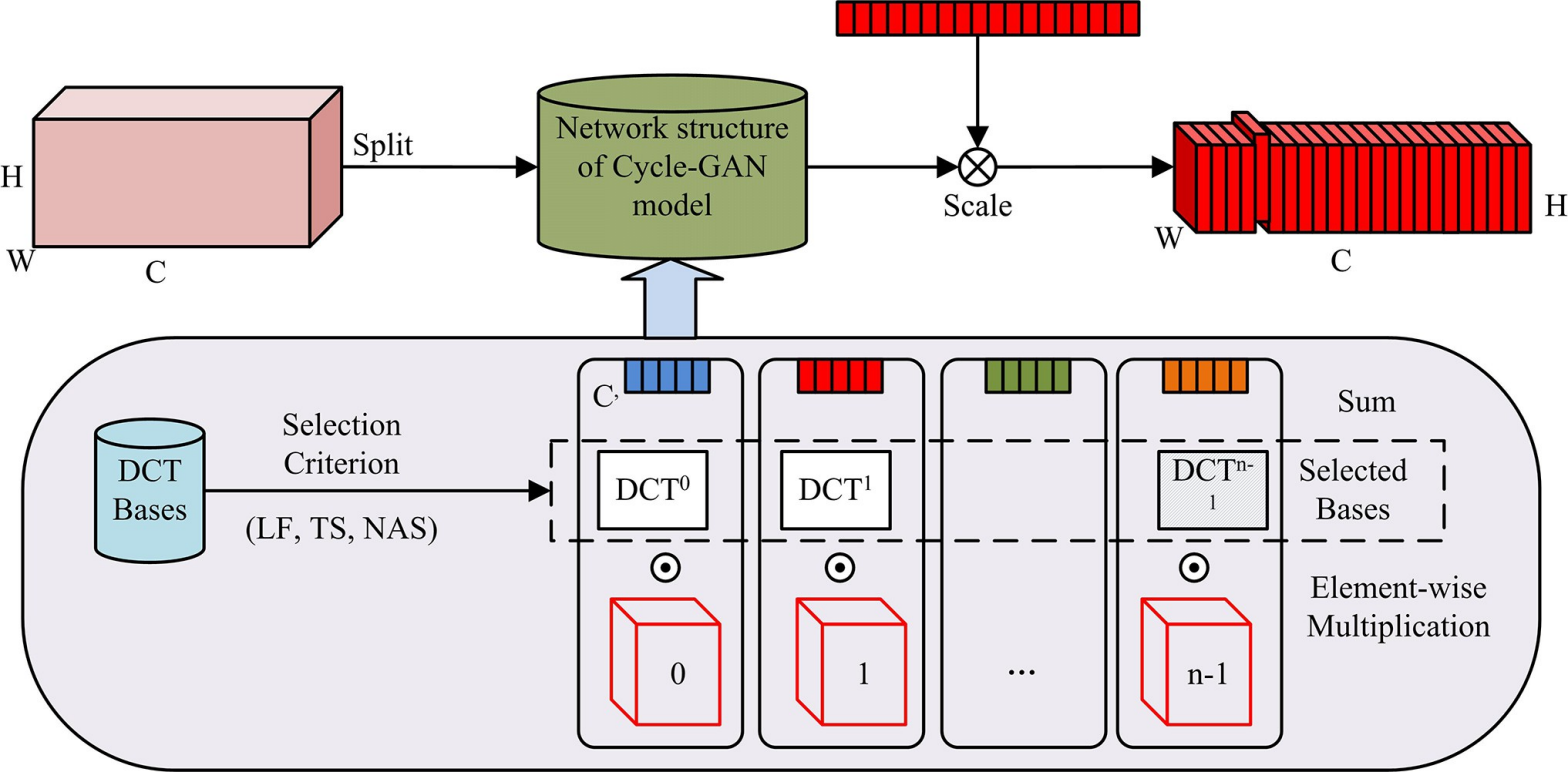
The specific architecture of the FcaNet-cycleGan style transfer method.

From the above equation, it can be seen that the original GAP method is extended to a multi-frequency component framework to enrich the compressed channel information more effectively. The process of integrating the attention mechanism with the GAN network specifically includes the following steps: first, use the cycle consistent adversarial network (CycleGAN) to extract the features of the illustration image; second, apply the attention mechanism to the extracted features and learn the weights. Focus on the parts of the image related to style transfer; then use the features extracted by the attention mechanism to perform style transfer and generate illustration images with a new style; finally combine the attention mechanism and GAN network to optimize the model parameters to achieve the goal of style transfer. The specific architecture of the FcaNet-cycleGan style transfer method is shown in Fig 6.

A method based on an improved cycle-consistent adversarial network (CycleGAN) was proposed, specifically for the task of style transfer from illustrations. The core innovation is the introduction of a novel loss function that can better capture the details and style features in illustrations. In addition, the network structure is adjusted, and a dedicated module is added to process the texture and color of the illustrations, thereby improving the accuracy and naturalness of the style transfer. A series of experiments are then conducted to prove that the constructed method can achieve a richer and more realistic style transfer effect while maintaining the original artistic style of the illustrations.

## 4. Performance test and application implementation of illustration image style transfer technology

To verify the superior performance of the improved cyclic consistency generative adversarial network constructed by the experiment in the style transfer of illustration images, the performance of the FcaNet-CycleGAN model in the illustration image style transfer system was analyzed. To avoid occasional errors due to different experimental environments, the dataset library and experimental settings are first introduced, and then the specific performance of the constructed system is systematically evaluated. The environmental parameters of the simulation experiment are shown as Table 1.

**Table 1:** Settings of related parameters.

Project	Choice
Processor	Intel(R)Xeon(R)CPUE7-4809V3@2.00GHz
Memory	8GB
Graphics card	NVIDIA GeForceRTX 3080
Operating system	Windows10
Programming language	Python3.7
Deep learning framework	Keras framework with Tensorflow as backend
Simulation tools	Simulink
GPU	RTX-2070
Data storage	MySQL
Data regression analysis platform	SPSS 26.0

**Fig 7:**
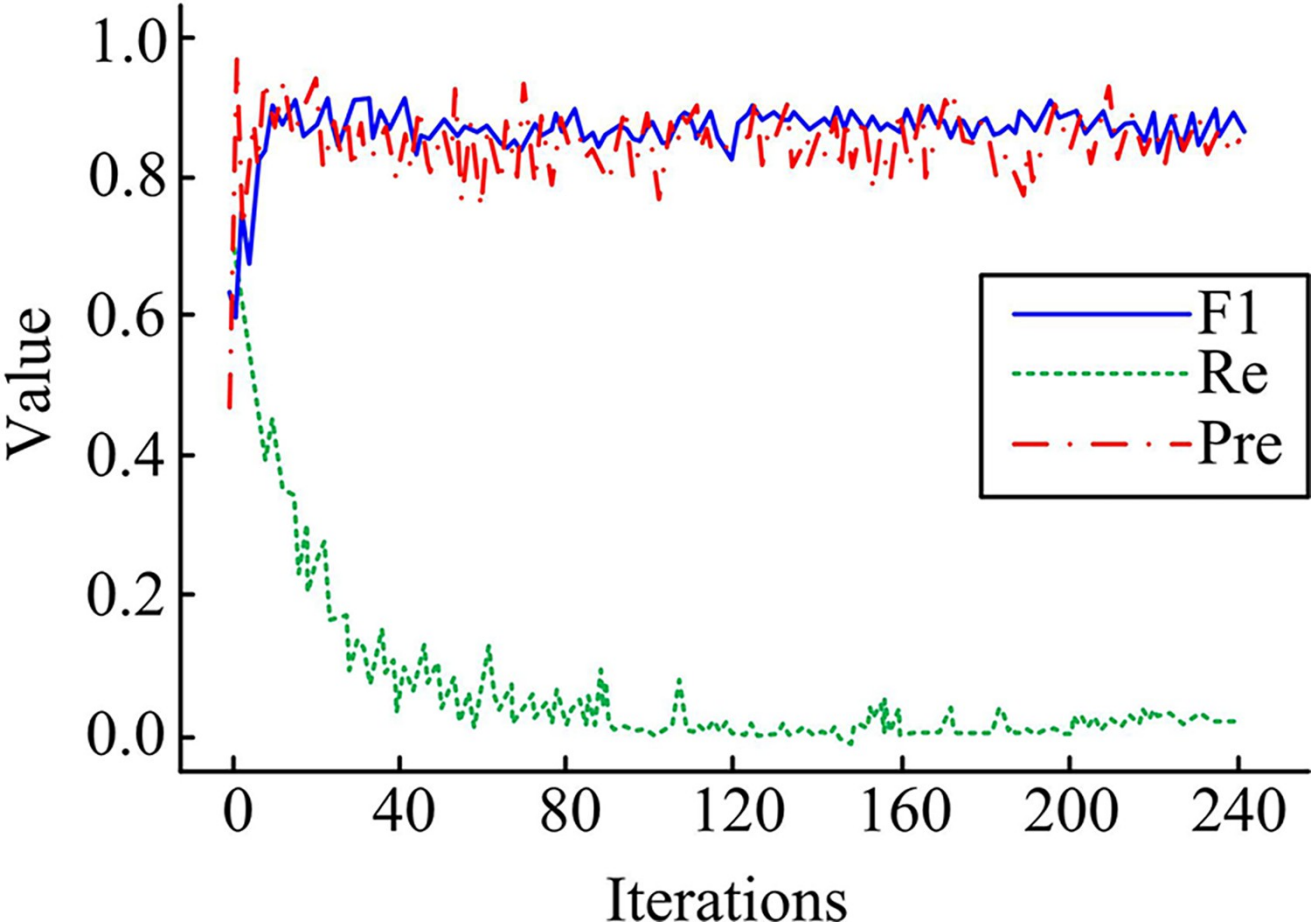
Comprehensive Re, F1 and Pre values of FcaNet-CycleGAN model.

The Monet2photo dataset and Horse2zebra dataset were selected as task datasets for the experiment. The Monet2photo dataset is a dataset composed of Monet’s paintings and real-world photos. It includes Impressionist style paintings by artist Claude Monet, as well as corresponding real-world photos. The goal of this dataset is to convert real-world photos into Monet style images. The Horse2zebra dataset is a dataset composed of images of horses and zebras. It includes photos of horses and zebras, both of which have different appearances and textures. The goal of this dataset is to convert images of horses into images of zebras, achieving cross species image style transfer. The Monet2photo dataset and the Horse2zebra dataset are two commonly used datasets in the field of image-to-image translation. They are not directly accessible. If you need them, you can find the data of the Monet2photo dataset through https://arxiv.org/abs/1703.01993; you can also find the data of the Horse2zebra dataset through https://arxiv.org/abs/1711.09020.. Next, the experiment selected cartoon image style transfer method using Attention Mechanism and Generative Adversarial Network (AM-GAN), nighttime vehicle detection day night image style transfer method using Data Augmentation and Generative Adversarial Network (DA-GAN) Comparison of performance between the pix2pix CycleGAN based Chinese ink painting reality interaction method based on deep learning and cyclic consistency GAN and the FcaNet Cycle GAN method constructed in the experiment [35–37]. Firstly, the comprehensive Re, F1, and Pre values obtained from the FcaNet Cycle GAN method running on two task datasets were analyzed, as shown in Fig 7.

**Fig 8:**
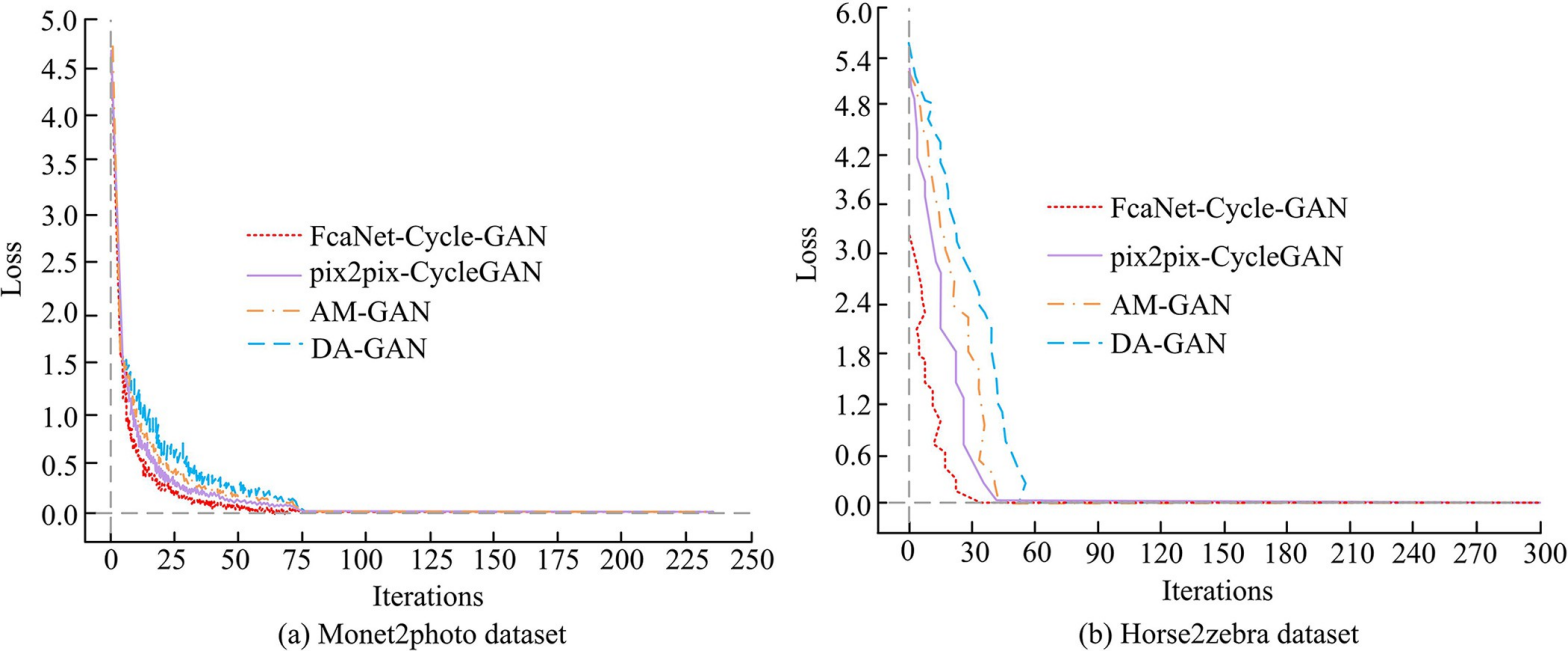
Changes in loss values for different algorithms.

From Fig 7, FcaNet Cycle GAN can start to have optimal solutions for the test items after training for the 160th time. At this time, the comprehensive Re, F1, and Pre values reached 0.122, 0.978, and 0.957, respectively, and the F1 value began to stabilize. A higher F1 value indicates that the model can cover a wide range of samples while maintaining accurate results. A higher Pre value indicates a lower error rate in recognizing illustration images. Overall, it indicates that the FcaNet Cycle GAN model has significant effectiveness and practicality. This is because in the improved cycle-consistent adversarial network, other technologies, such as the Feature Attention mechanism, may be introduced to further improve the accuracy of feature extraction and the quality of style transfer. Compare the loss function values obtained from training four algorithms on two datasets, as shown in Fig 8.

**Fig 9:**
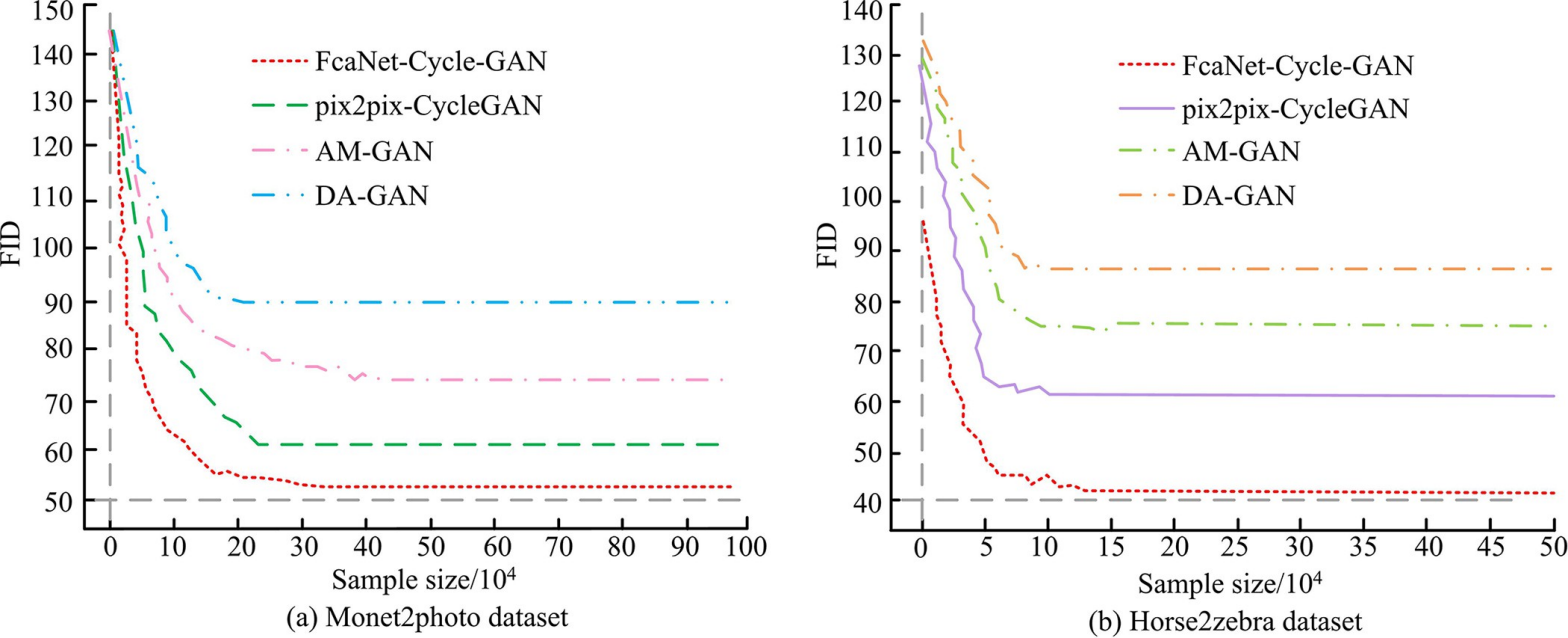
Changes in FID values of different algorithms.

Fig 8(A) shows the variation curves of the loss functions of the four algorithms running on the Monet2photo dataset. It can be found how the loss function of the four algorithms gradually decreases and tends to stabilize throughout the training process. When the system iterates to 72 times, the loss function value of the FcaNet-CycleGAN method begins to have a minimum value, which tends to the target value of 0.00 and remains unchanged in the future, while the loss function values of the pix2pix-CycleGAN, DA-GAN and AM-GAN algorithms are still changing, and the number of iterations reaching the steady state is significantly larger. Fig 8(B) shows the change in the value of the loss function on the Horse2zebra dataset. When the system iterates to 34 times, the loss function value of the FcaNet-CycleGAN method begins to stabilize, while when the loss function values of pix2pix-CycleGAN, DA-GAN and AM-GAN algorithms tend to be stable, the number of iterations of the corresponding system is 48, 57 and 51, respectively. The comparison shows that the loss function value of FcaNet-CycleGAN decreases rapidly and tends to a low stable value, which indicates that FcaNet-CycleGAN converges quickly and has good stability in the learning process. The rapid decline of the loss function and the low stable value prove the efficiency and effectiveness of the method. This is similar to the convergence property of the Adams method. The Adams method can enhance the stability of the network and reduce the oscillation of the loss function in the later period. Then, the FID values of different algorithms under the operation of the four algorithms are compared, as shown in Fig 9.

**Fig 10:**
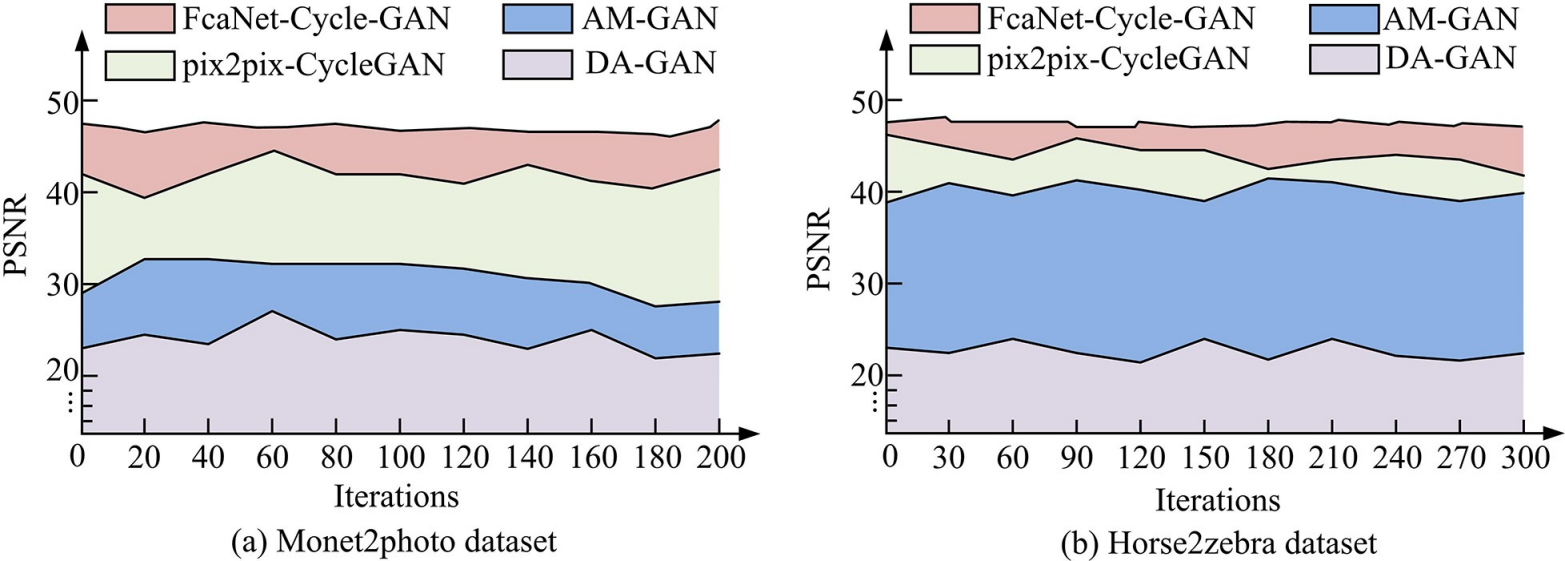
Comparison of PSNR values of different algorithms.

In Fig 9, the Fréchet Inception Distance (FID) value is an important indicator to measure the quality of the generated image, and the lower the value, the closer the feature distribution between the generated image and the real image, that is, the higher the image quality. Fig 9(A) shows the change in FID values on the Monet2photo dataset. As experimental samples increase, FcaNet-CycleGAN’s curve is significantly lower than that of the other three algorithms. When the sample size reaches 34×10^4^, the FcaNet-CycleGAN model has a minimum FID value of 51.24, while the FID values of the other three algorithms are larger than those of the research method. Fig 9(B) shows the change of the FID value curve on the Horse2zebra dataset. It can be observed that the FID curve of FcaNet-CycleGAN shows a steady downward trend from left to right, and finally stabilizes at a very low value, which is infinitely close to 40.00, but the FID curves of the other three algorithms also show a downward trend, but the decline rate is slower and the final stable value is higher. This means that with the deepening of model training, the image quality generated by style transfer under FcaNet-CycleGAN is improved, and the illustration image is more visually realistic and high-quality, and the difference between the illustration image and the real image is very small. This is because introducing the attention mechanism and optimizing the loss function can improve the model’s ability to capture style features, thereby obtaining lower results in FID values. Then, the average Peak Signal-to-Noise Ratio (PSNR) repaired by the four algorithms for the two datasets was analyzed in Fig 10.

**Fig 11:**
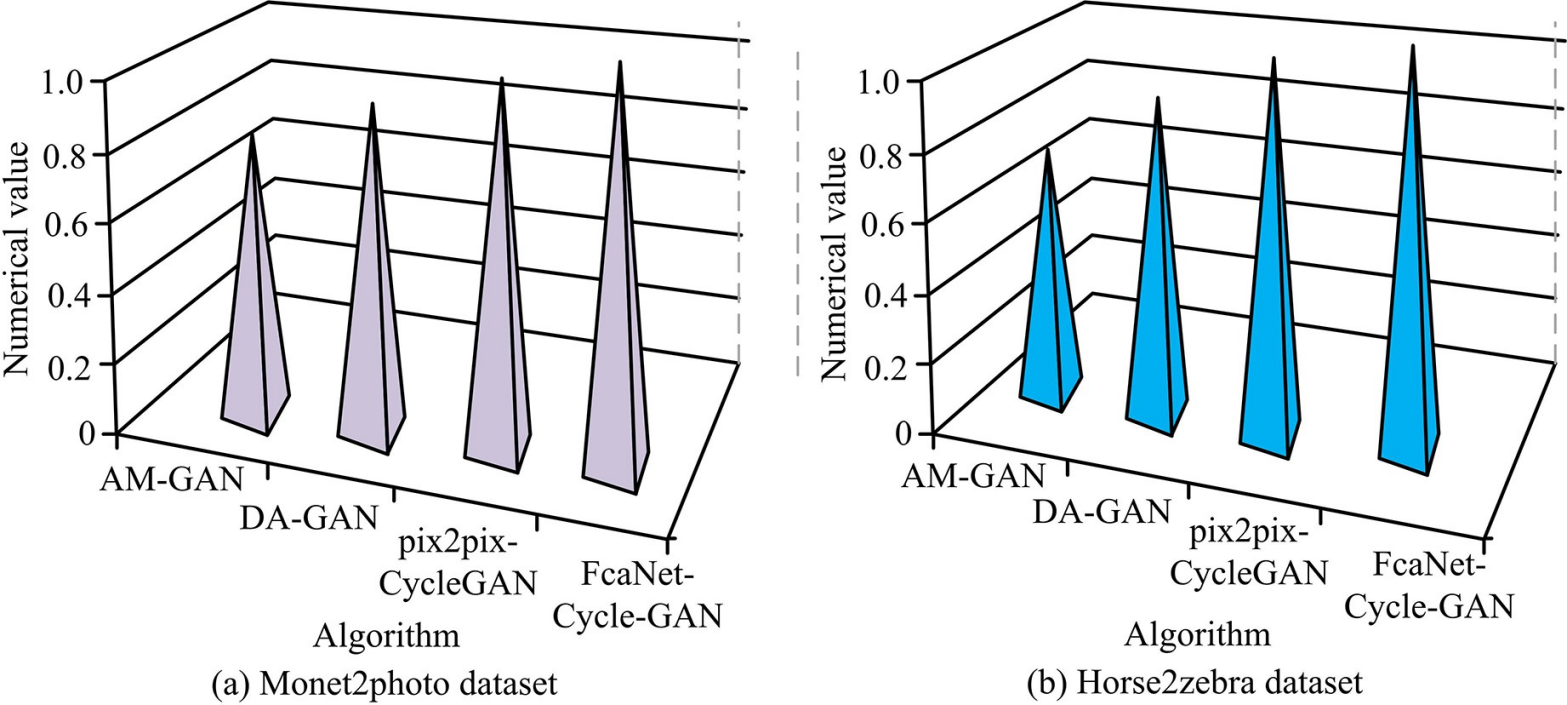
Comparison of SSIM values of different algorithms.

In Fig 10, PSNR is a common measure of image quality to evaluate the quality of image reconstruction. Fig 10(A) shows the change of PSNR values of the four algorithms on the Monet2photo dataset. PSNR of the FcaNet-CycleGAN method is higher throughout the whole system iteration process, and the fluctuation is small in the whole process, and the value is as high as 47.89dB. Among the other three algorithms, the pix2pix-CycleGAN method has a higher PSNR value of 45.77dB. Fig 10(B) shows the PSNR values on the Horse2zebra dataset. With the iterative change of the system, the PSNR value of FcaNet-CycleGAN reached a new height, with a value as high as 48.45dB, and remained stable continuously. The other three methods showed a relatively stable state, but all of them were smaller than the FcaNet-CycleGAN method. The above results illustrate the significant advantages of the FcaNet-CycleGAN method in improving the style transfer quality of illustration images, and its high PSNR value indicates the excellent performance of the method in image reconstruction and style preservation. Then, the SSIM values under the operation of the four algorithms are compared, and the results are shown in Fig 11.

**Fig 12:**
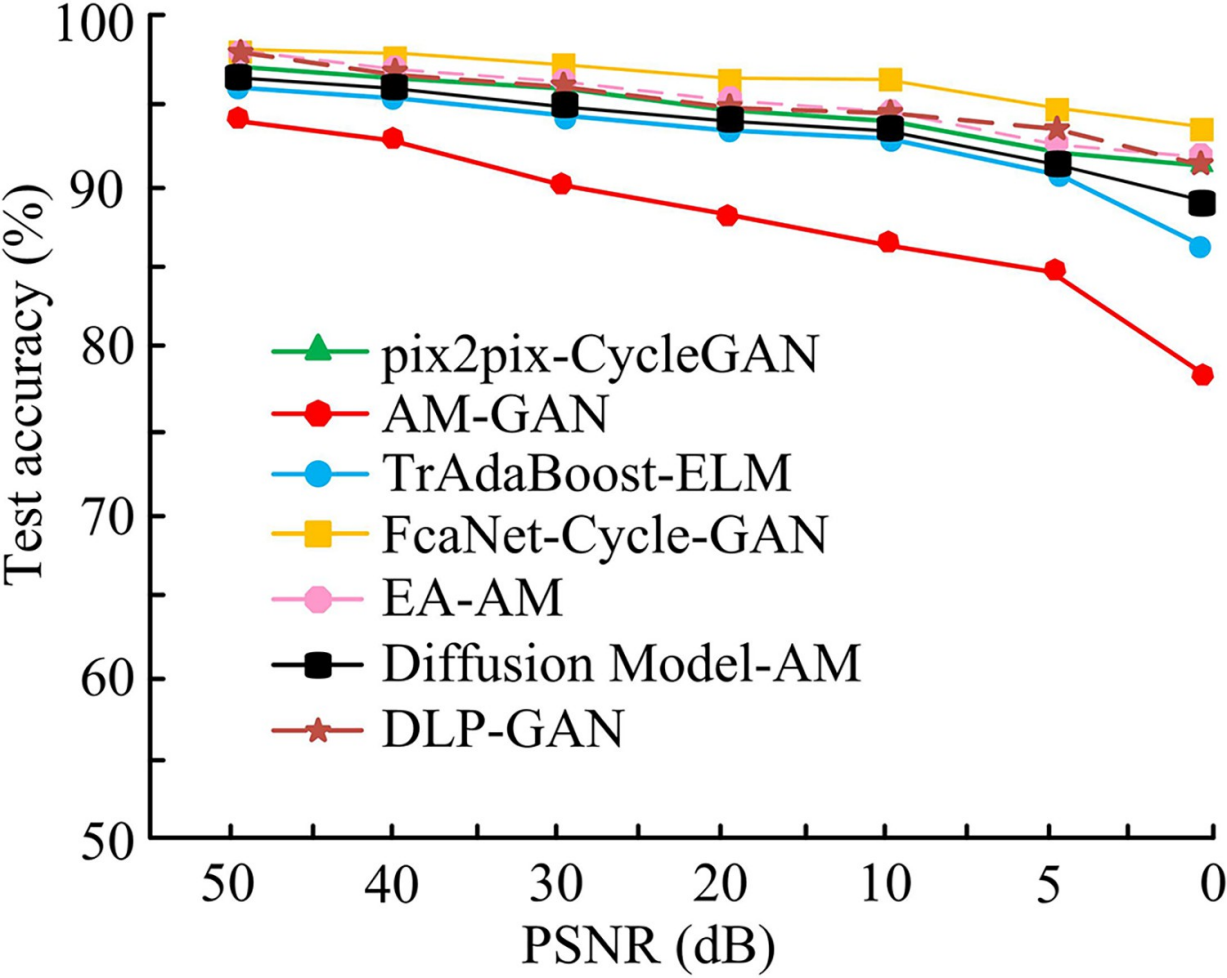
Schematic diagram of the accuracy of different network models changing with the signal-to-noise ratio.

Fig 11(A) shows the change in SSIM values on the Monet2photo dataset. The maximum SSIM value of the FcaNet-CycleGAN method is 0.9547, which is higher than that of pix2pix-CycleGAN of 0.854, DA-GAN of 0.789 and AM-GAN of 0.698. Fig 11(B) shows the change in SSIM values on the Horse2zebra dataset. The maximum SSIM value of FcaNet-CycleGAN is 0.9841, higher than that of pix2pix-CycleGAN (0.921), DA-GAN (0.756), and AM-GAN (0.602). Taken together, the top of the FcaNet-CycleGAN tapered bar is close to the upper bound of the chart, and its SSIM value is significantly close to 1, which indicates that the method performs well in maintaining the structural characteristics of the original image. The excellent performance of FcaNet-CycleGAN may be attributed to its improved cyclic consistency adversarial network structure, which improves the structure retention ability of the image through more effective loss function and network design, so that the image after style migration can maintain the original content structure while obtaining good visual effects. To further verify the results, the study introduced an improved generative adversarial network image translation method based on evolutionary algorithms and attention mechanisms (EA-AM) [38], a modern Chinese landscape photo drawing method based on dense fusion modules and generative adversarial networks (Draw Modern Chinese Landscape Photos with Generative Adversarial Network, DLP-GAN) [39], and an image semantic guidance method based on diffusion model and attention (Diffusion model and attention mechanism, Diffusion Model-AM) [40] to compare their performance with the other four network models (pix2pix-CycleGAN, AM-GAN, TrAdaBoot-ELM and FcaNet-Cycle-GAN). The results are shown in Fig 12.

**Fig 13:**
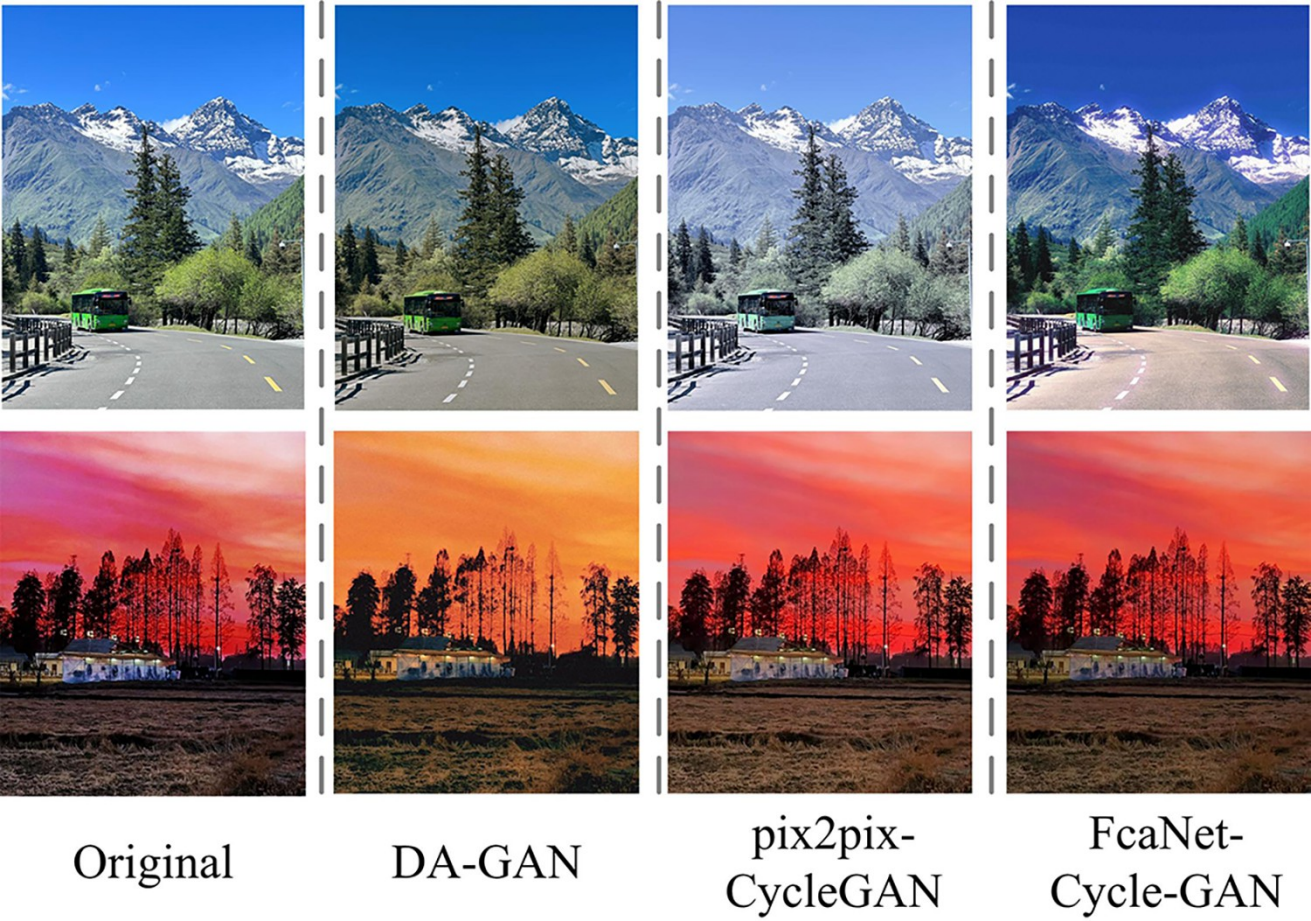
The actual training effect of four algorithms on original photo data (All images are original images taken by the author).

In Fig 12, the accuracy of style transfer to illustration images decreases with the decrease of PSNR values for the four different network models. However, the accuracy of the FcaNet-CycleGAN model decreases more slowly. Until the PSNR value was reduced to 0, the accuracy of the FcaNet-CycleGAN model was greater than 95.00%. As the PSNR value decreases, the accuracy of the EA-AM algorithm, the DLP-GAN algorithm, and the Diffusion Model-AM algorithm has been decreasing, and has always been lower than the FcaNet-Cycle-GAN model; among them, the accuracy of the Diffusion Model-AM algorithm is much lower than that of the FcaNet-Cycle-GAN model. The FcaNet-Cycle-GAN model is more resistant to noise and can accurately achieve style transfer of illustrations. The FcaNet-Cycle-GAN model, pix2pix-CycleGAN and TrAdaBoost-ELM with better performance were actually trained on the Monet2photo dataset, and the style similarity between the target style and the generated image was selected to compare the migration effects of different methods. The obtained illustration style migration effect is shown in Fig 13.

**Fig 14:**
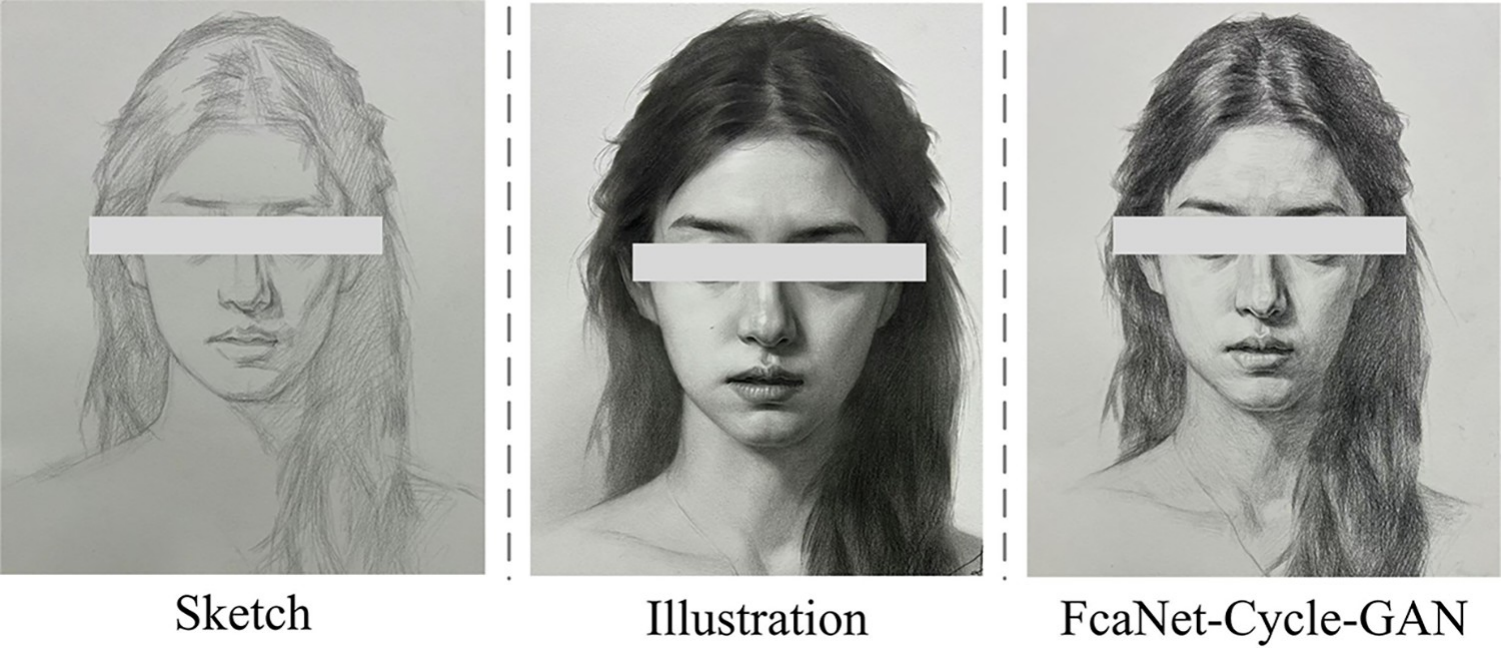
Style transfer effect between illustrations and images of different artistic styles (All images are original creations created by the author).

In Fig 13, the migration results of all models retain the contour structure of the original image, but FcaNet-CycleGAN is more brilliant in color and the line features are more obvious, which not only retains the clear texture information, but also distinguishes the boundary colors between different objects in the image and the color tone is more prominent. Finally, the FcaNet-CycleGAN model was used to transfer the style of illustrations and images of different art styles, and the specific effect is shown in Fig 14.

Fig 14 visually illustrates the efficiency of the FcaNet-CycleGAN method in performing the task of transferring the style of illustration images. Through a series of image comparisons, the chart shows that the images processed by FcaNet-CycleGAN can be combined with different artistic styles. Finally, to analyze the difference between the improved model and the unimproved model and illustrate the effectiveness of the internal modules, the FcaNet-Cycle-GAN method, Cycle-GAN module, FcaNet model, and RFA model were compared in terms of performance indicators such as SSIM and PNSR. 50 target users (including non-professionals and domain experts) were selected to conduct subjective evaluation and scoring of the effects before and after image style transfer. The results are shown in Table 2.

**Table 2:** Comparison of ablation experiment results.

Module/Method	SSIM	PNSR	MSE	Time consumption	Score
RFA model	0.901	28.218	0.1174	0.85s	79.5
FcaNet model	0.911	28.235	0.1146	0.78s	80.2
Cycle-GAN module	0.921	29.402	0.0935	0.71s	83.4
FcaNet-Cycle-GAN method	0.954	35.948	0.0125	0.52s	91.6

As can be seen from Table 2, the SSIM, PNSR and MSE values of the proposed FcaNet-Cycle-GAN method are 0.954, 35.948 and 0.0125, respectively, which are much smaller than the other three methods. Taking the Cycle-GAN module as an example, the SSIM, PNSR, MSE and time consumption values of this method are 0.921, 29.402, 0.0935 and 0.71s respectively. By comparison, the mean square error of the FcaNet-Cycle-GAN method is smaller, and the SSIM value and PNSR value are greater than those of other methods. In addition, the experiment selected running time to analyze the efficiency of the algorithm, and selected frame rate to analyze the complexity of the algorithm. Then, four models were used to process 100 images. The average running time of the FcaNet-Cycle-GAN model was only 8.51s; the frame rate was as high as 108.95 frames per second. This also shows that the image migration effect obtained by the FcaNet-Cycle-GAN method is better, and the quality of the constructed model is better.

The subjective evaluation results show that the improved FcaNet-Cycle-GAN achieved a higher score in the style transfer task. Specifically, the improved model performed better in preserving details, and the target users generally believed that the improved model better balanced the preservation of content and the fusion of style in style transfer, which shows that the proposed method effectively improves the visual quality of style transfer. In order to demonstrate the influence of different attention mechanisms on illustration image style transfer tasks, an ablation experiment was designed to compare the performance differences between the proposed attention mechanisms and other types of attention mechanisms. The experimental results are shown in Table 3.

**Table 3:** Results of ablation experiment.

Types of attention mechanisms	Accuracy rate (%)	Recall rate (%)	F1 score	Lost value
Inattentive mechanism	85.2	82.4	0.837	0.042
Channel attention	87.6	85.1	0.860	0.039
Spatial attention	88.1	86.3	0.869	0.037
Self attention Multiscale attention	89.2	87.4	0.880	0.036
Study proposed attention mechanism	90.1	88.5	0.890	0.035
Types of attention mechanisms	92.3	90.5	0.910	0.032

As can be seen in Table 3, the introduction of attention mechanism significantly improves the performance indicators of the model. The channel attention and spatial attention models improved accuracy rates by 2.4% and 2.9%, respectively, and recall rates by 3.7% and 3.9% over the baseline model, showing that focusing on specific features improves model performance. The self-attention and multi-scale attention models further optimized performance with accuracy of 89.2% and 90.1%, recall rates of 87.4% and 88.5%, respectively, and F1 scores and loss values also showed further improvements in model performance. The attention mechanism proposed in the study showed the best performance, the accuracy increased to 92.3%, the recall rate was 90.5%, the F1 score was as high as 0.910, and the loss value decreased to 0.032, indicating that the attention mechanism considering the channel and spatial characteristics has a significant advantage in the style transfer task. If the attention mechanism proposed in the study is replaced with other types of attention mechanisms, the inattentional mechanism results in a significant drop in accuracy because it fails to highlight key features in the image, while channel attention and spatial attention, while capable of improving performance, are less effective than multi-scale attention because they ignore global or local style features. Although self-attention mechanisms can capture long-distance dependencies, they are not efficient in style transfer tasks due to high computational costs and insensitivity to small-scale data. Although multi-scale attention can consider features of different scales at the same time, the increase of computational complexity leads to longer training time and increased model complexity. In contrast, the proposed attention mechanism combines the advantages of multiple attention mechanisms to capture and transfer style features more precisely, thus achieving higher accuracy and lower loss values in style transfer tasks. FcaNet-Cycle-GAN was compared with other recent CycleGan-based methods, and the indicators were normalized. The final results were shown in Table 4.

**Table 4:** Comparison of results based on CycleGAN method.

Methods	Style consistency	Detail retention	Run time (s)	Overall evaluation
CycleGAN	0.90	0.85	0.95	0.88
CycleGAN++	0.92	0.88	1.05	0.90
Attention-CycleGAN	0.93	0.90	1.10	0.92
Dense-CycleGAN	0.94	0.92	1.20	0.93
FcaNet-Cycle-GAN	0.98	0.96	1.08	0.97

In Table 4, FcaNet-Cycle-GAN method is superior to other methods based on CycleGAN in terms of style consistency and detail retention, and also has good performance in terms of running time, with the highest overall evaluation, which verifies its effectiveness and superiority in the task of illustration image style transfer. In order to demonstrate the performance of FcaNet-Cycle-GAN method and other Cyclegan-based methods in statistical analysis, these methods were statistically analyzed in this study. The statistical analysis table is shown in Table 5.

**Table 5:** Statistical analysis table.

Methods	T-test (*p* value)	ANOVA (*p* value)	Correlation (r value)	References
CycleGAN	0.05	<0.001	0.75	C. Y. Chung and S. H. Huang [37]
CycleGAN++	0.04	<0.001	0.80	Y. Xue et al. [38]
Attention-CycleGAN	0.03	<0.001	0.85	X. Gui et al. [39]
Dense-CycleGAN	0.02	<0.001	0.88	H. Chefer et al. [40]
FcaNet-Cycle-GAN	<0.001	<0.001	0.92	This study

In Table 5, the *p*-values of the t-test show that all improved methods show statistically significant differences compared to the baseline CycleGAN, with FcaNet-Cycle-GAN having the lowest *p*-value, indicating its most significant improvement compared to the baseline. The *p* values of ANOVA were all less than 0.001, indicating significant performance differences between different methods. FcaNet-Cycle-GAN has the highest r value, reaching 0.92, indicating that its performance is most closely related to the ideal state, showing the strongest prediction ability and consistency. FcaNet-Cycle-GAN outperforms other Cyclegan-based methods in terms of style consistency, detail retention, and overall performance.

## 5. Conclusion

To improve insufficient detail retention and inaccurate style transfer in the traditional CycleGAN in the style transfer of illustration images, a new method combining FcaNet and improving the CycleGAN network was proposed. This method fuses the improvement of the feature AM-GAN, and better retains the details and texture of the image while maintaining the original illustration content. The data showed that when the system iterated to 34 times on the Horse2zebra dataset, the loss function value of the FcaNet-CycleGAN method began to stabilize, while the loss function values of the pix2pix-CycleGAN, DA-GAN, and AM-GAN algorithms tended to be stable, and the iterations of the corresponding system were 48, 57 and 51, respectively. When running on the Monet2photo dataset, when the sample size reached 34×104, the FcaNet-CycleGAN model had a minimum FID value of 51.24, while the FID values of the other three algorithms were larger than those of the research method, and the overall fluctuation of the PSNR value of the FcaNet-CycleGAN method was small, and the value was stable at 47.89dB. The FcaNet-CycleGAN model was applied to the Monet2photo dataset for training, and the obtained image not only retained clear texture information, but also distinguished the boundary colors between different objects in the image, and the color tone was more prominent. In general, the FcaNet-CycleGAN model shows great potential in illustration image style transfer, and significantly improves the consistency of style transfer results. Future work will focus on further improving the model generalization capabilities to accommodate a wider range of art styles and illustration types, as well as exploring more effective training strategies and network structures to continuously optimize the performance and quality of style transfer.

## Supporting information

S1 Dataset The data in Fig 7. (DOC)
