# Supplementary material for: Illustration image style transfer method design based on improved cyclic consistent adversarial network
Source: PLoS One. 2025 Jan 14;20(1):e0313113. doi: 10.1371/journal.pone.0313113 (PMC11981539; doi:10.1371/journal.pone.0313113)
Supplement: S1 Dataset — (DOC) [file pone.0313113.s001.doc]

**The data in Figure 7**

| Iterations | FcaNet-Cycle-GAN model | | |
| --- | --- | --- | --- |
|  | Re value | F1 value | Pre value |
| 80 | 0.134 | 0.976 | 0.954 |
| 160 | 0.122 | 0.978 | 0.957 |
| 240 | 0.128 | 0.975 | 0.960 |

**The data in Figure 8**

| Sample size/10^4^ | Monet2photo dataset | | | |
| --- | --- | --- | --- | --- |
|  | FcaNet-Cycle-GAN | pix2pix-CycleGAN | AM-GAN | DA-GAN |
| 10 | 64.14 | 79.82 | 91.87 | 100.02 |
| 20 | 55.13 | 65.56 | 80.09 | 90.01 |
| 34 | 51.24 | 60.94 | 77.63 | 90.00 |
| Sample size/10^4^ | Horse2zebra dataset | | | |
|  | FcaNet-Cycle-GAN | pix2pix-CycleGAN | AM-GAN | DA-GAN |
| 5 | 49.87 | 65.03 | 89.42 | 104.12 |
| 10 | 45.11 | 61.98 | 75.16 | 86.04 |
| 15 | 41.46 | 61.599 | 74.78 | 86.04 |

**The data in Figure 9**

| Loss | Monet2photo dataset | | | |
| --- | --- | --- | --- | --- |
|  | FcaNet-Cycle-GAN | pix2pix-CycleGAN | AM-GAN | DA-GAN |
| 25 | 0.23 | 0.29 | 0.47 | 0.69 |
| 50 | 0.08 | 0.11 | 0.18 | 0.31 |
| 72 | 0.00 | 0.05 | 0.10 | 0.15 |
| 100 | 0.00 | 0.00 | 0.00 | 0.00 |
| Loss | Horse2zebra dataset | | | |
|  | FcaNet-Cycle-GAN | pix2pix-CycleGAN | AM-GAN | DA-GAN |
| 30 | 0.07 | 0.56 | 1.76 | 2.74 |
| 34 | 0.00 | 0.38 | 0.59 | 2.45 |
| 60 | 0.00 | 0.00 | 0.00 | 0.00 |

**The data in Figure 10**

| Iterations | Monet2photo dataset | | | |
| --- | --- | --- | --- | --- |
|  | FcaNet-Cycle-GAN | pix2pix-CycleGAN | AM-GAN | DA-GAN |
| 100 | 46.58 | 42.01 | 32.04 | 25.19 |
| 200 | 57.92 | 42.68 | 28.00 | 22.86 |
| Iterations | Horse2zebra dataset | | | |
|  | FcaNet-Cycle-GAN | pix2pix-CycleGAN | AM-GAN | DA-GAN |
| 150 | 47.19 | 44.18 | 39.07 | 24.00 |
| 300 | 47.19 | 42.00 | 39.98 | 22.79 |

**The data in Figure 11**

| Max SSIM value | Monet2photo dataset | | | |
| --- | --- | --- | --- | --- |
|  | FcaNet-Cycle-GAN | pix2pix-CycleGAN | DA-GAN | AM-GAN |
|  | 0.955 | 0.854 | 0.789 | 0.698 |
| Max SSIM value | Horse2zebra dataset | | | |
|  | FcaNet-Cycle-GAN | pix2pix-CycleGAN | DA-GAN | AM-GAN |
|  | 0.984 | 0.921 | 0.756 | 0.602 |

**The data in Figure 12**

| PSNR | Test accuracy (%) | | | | |
| --- | --- | --- | --- | --- | --- |
|  | FcaNet-Cycle-GAN | EA-AM | pix2pix-CycleGAN | DA-GAN | AM-GAN |
| 10 | 96.47 | 94.67 | 94.01 | 92.87 | 96.51 |
| 30 | 97.08 | 96.48 | 95.04 | 94.14 | 90.00 |
| 50 | 98.00 | 97.98 | 97.03 | 96.00 | 94.05 |
